# Interventions influencing patient-reported outcomes (PROs) response rates in cancer: a scoping review

**DOI:** 10.1007/s11764-025-01801-9

**Published:** 2025-04-15

**Authors:** Nada Al-Antary, Samantha Tam, Suma Alzouhayli, Theresa M. Zatirka, Michael Ryan, Steven S. Chang, Benjamin Movsas, Eric Adjei Boakye

**Affiliations:** 1 Department of Public Health Sciences, Henry Ford Health, One Ford Place Detroit, MI 48202 USA; 2 Department of Otolaryngology – Head and Neck Surgery, Henry Ford Health, Detroit, MI USA; 3 Henry Ford Health + Michigan State University Health Sciences, Detroit, MI USA; 4 Wayne State University School of Medicine, Detroit, MI USA; 5 Henry Ford Cancer, Henry Ford Health, Detroit, MI USA; 6 Department of Supportive Oncology, Henry Ford Health, Detroit, MI USA; 7 Department of Radiation Oncology, Henry Ford Health, Detroit, MI USA; 8 Department of Epidemiology and Biostatistics, Michigan State University College of Human Medicine, East Lansing, MI USA

**Keywords:** Patient-reported outcomes (PROs), Patient-reported outcome measures (PROMs), Completion rate, Cancer survivors, Real-world setting

## Abstract

**Purpose:**

Despite the emerging evidence around patient-reported outcome measures (PROMs) monitoring benefits in oncology, completion rates remain low due to numerous multi-level barriers. This review summarizes existing literature on interventions employed to improve PROMs response rates in routine practice among patients with cancer.

**Methods:**

PubMed database was used to perform a literature search of articles published between 2000 and 2022. Articles were included if they focused on PROMs implementation in non-clinical trial setting and reported results on methodologies and their influence on response rates.

**Results:**

A total of 495 abstracts were screened for eligibility, and 14 articles that met the inclusion criteria were included. PROMs mode of administration varied between electronic only (four studies, 28.6%), paper only (two studies, 14.3%), electronic-paper (six studies, 42.9%), and electronic-telephone (two studies, 14.3%). Reminder systems, using electronic, paper, or in-person, were implemented in 12 studies (85.7%). Different strategies of initial recruitment, aiming to enhance patients’ PROM engagements, were outlined in five studies (35.7%).

**Conclusion:**

Multiple interventions were implemented to improve PROMs completion rates. Mode of questionnaire administration, reminder systems, patient education on benefits of PROMs, and clinical staff involvement were shown to be effective in increasing the overall completion rate.

**Implications for Cancer Survivors:**

This review provides a summary for researchers and clinicians on the current practice of PROMs implementation, thus creating a framework for the impact of different methodologies on patient’s response rate for better monitoring of recurring symptoms, including long-term side effects, emotional distress, and changes in health-related quality of life.

**Supplementary Information:**

The online version contains supplementary material available at https://doi.org/10.1007/s11764-025-01801-9.

## Introduction

Despite the advancements in cancer multidisciplinary and multimodal treatment approaches and their positive impact on overall survival and cure rates, patients with cancer remain at high risk of various generalized and unspecific symptoms. These symptoms significantly affect patients’ function and quality of life (QoL), which can be attributed either to the cancer itself or to the adverse effects of the treatment [1, 2]. Moreover, current day practice relies heavily on the physician’s interpretation of the patient’s health status, which may not be completely representative of the patient’s holistic experience, keeping the clinician’s interpretation of the patient’s health status as the primary focus of patient care [3]. Elevation of the patient’s voice is essential in clinical assessments which traditionally center around provider generated and interpreted data. Patient-reported outcomes (PROs) have been suggested to be as important in clinical assessments as traditional vital signs [4]. PROs have been demonstrated to complement physician assessments [5].

PROs offer a window for patients to report their health status and independently share their own experience by measuring patients’ perceptions of their physical and mental well-being [6, 7]. Sharing PROs data with physicians can help identify patients experiencing persistent, deteriorating, or severe symptoms and thus map out potential opportunities for interventions and facilitate shared decisions between patients and their healthcare team [8, 9]. PROs provide a systematic approach in monitoring symptoms among patients with cancer and aid in general health assessment and symptom management, thus capturing symptoms early and potentially enhancing QoL [10–12]. PROs may therefore allow for enhanced patient-provider communication and patient satisfaction for more personalized cancer care [13, 14]. In addition, PROs monitoring reduces hospitalizations and emergency room visits compared to usual care [15, 16]. A recent trial reported that monitoring of PROs in cancer care and acting upon the symptoms could be associated with increased survival and better prognosis [17]. These benefits might be attributed to early recognition of patients who require referrals and adequate management of their symptoms [14, 17, 18].

Despite the benefits of PRO monitoring, completion rates of patient-reported outcome measures (PROMs) in clinical practice remain low (< 50%) across multiple settings [19–24]. For PROs benefits to be realized, patients have to complete PROMs. Multiple interventions can be employed to promote a more inclusive and generalizable utilization of PROs. Efforts may focus on a patient-centered design to address the most common barriers impeding patients from completing PROs. This might include using reminder systems, personnel to assist patients, providing both paper and electronic options, and translating PROs to various languages. The purpose of this scoping review was to summarize literature on interventions employed to improve PROs response rates in real-world or non-clinical trial research setting among patients diagnosed with cancer. Articles conducted in non-clinical trial research setting were targeted as an attempt to reflect the closest conditions to real-world setting. As PRO implementation in routine care faces different challenges hindering healthcare systems and patients from adopting PROs as part of clinical practice, more specific data is needed to reflect on the different real-world approaches to facilitate utilization of these tools.

## Methods

This scoping review did not require institutional review board because it was not a human subject research, and it used only published articles. A scoping review design was adopted for this study considering the insufficient evidence around effective strategies to maximize PROM response rates among diverse populations with different cancer diagnoses in real-world/non-clinical trial settings. This design allows summarizing current available data corresponding to the objective of this study. The Preferred Reporting Items for Systematic reviews and Meta-Analyses extension for Scoping Reviews (PRISMA-ScR) reporting guideline was followed while conducting this review [33].

### Search strategy

We iteratively developed the search strategy in consultation with a health sciences librarian from our institution. Studies were identified by conducting a literature review of the PubMed database using search terms and MeSH headings that included key words related to *cancer*, *patients*, *PROs*, *patient reported outcomes*, and *response rates*. The complete search terms used are presented in Supplemental Table 1 per PRISMA-ScR reporting guidelines. A second health sciences librarian peer-reviewed the searches. We conducted our search between October 1 and 18, 2022.

### Study eligibility and selection

Inclusion and exclusion criteria were created to assess study eligibility. Studies were included if they (1) focused on PROs implementation in patients with cancer as real-life clinical application only; herein, we targeted studies with cross-section, prospective, or retrospective design conducted for either routine implementation of PRO program or non-clinical trial research as the closest representation of real-world setting, (2) included a population of patients with cancers or survivors of less than 5 years only, (3) used an intervention to influence PROM response rate, (4) reported results of the intervention, (5) were published in English, (6) were published between January 2000 and October 2022, and (7) had accessible full texts. Studies that were aiming to develop or validate their own PRO tools without using interventions to influence the response rate, those that used PROs in patients without cancer or in clinical trials, in addition to case reports, case series, study protocols, editorials, dissertations, and conference abstracts were all excluded. Initially, titles and abstracts were screened by two authors (N.A, S.A). For relevant abstracts, full text articles were then screened to further evaluate eligibility. Finally, the articles included were reviewed by two authors (N.A, S.A) and a third author (E.A.B) to eliminate any discrepancy and ensure consensus.

### Data extraction

For articles that met the inclusion criteria, a table was designed and approved by all reviewers to guide the data extraction. The following data points were extracted: authors and journal, year of publication, methodology, setting and population, study sample size, patients’ demographics (age, gender, race), cancer type, PROMs instrument, survey frequency, intervention implemented to influence response rate, and results of the intervention. Not reported “NR” was used to identify any missing data. Data extraction was carried out by two authors, and the final table design and content were edited to ensure agreeability between all authors.

### Quality assessment

Study quality was assessed using the National Heart, Lung, and Blood Institute Study Quality Assessment Tool for Observational Cohort and Cross-Sectional Studies [34]. The criteria included 14 questions that guided detailed examination of key concepts for evaluation of studies’ internal validity and overall reliability. This includes assessing elements such as the research question, study population, sample size, timeframe, outcome measures, and statistical analysis. By systematically examining these factors, two authors (N.A. and E.A.B) independently labeled each study into one of three quality ratings (Good, Fair, or Poor), and a discussion was conducted in case of discrepancies to reach a final rating.

## Results

### Baseline characteristics of included studies

**Fig. 1:**
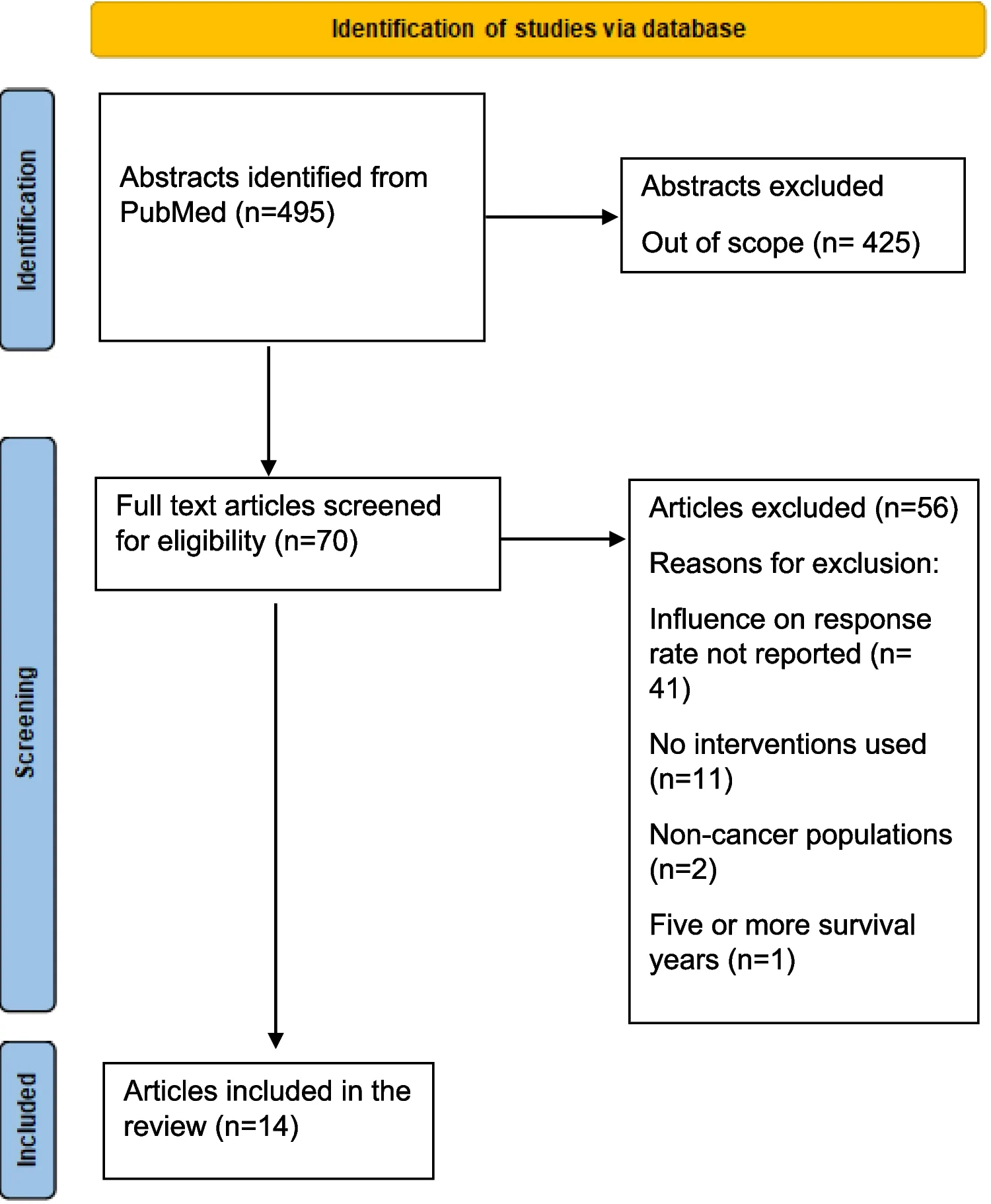
PRISMA flow diagram of included studies

A total of 495 abstracts identified through the electronic PubMed database literature search were screened for eligibility (Fig. 1). After excluding abstracts that were out of scope, 70 full text articles were considered for further review. Articles were excluded for different reasons, most commonly due to lack of reporting on the effect of the intervention on the response rate (41 articles) or lack of use of any intervention (11 articles). Full-text articles screening yielded 14 articles that met the inclusion criteria and were included in this study. All studies included in the final review were published between 2012 and 2022 (Table 1). Among them, six studies were conducted in the USA [28, 35–39], three in Denmark [40–42], two in the UK [21, 43], two in the Netherlands [44, 45], and one in Republic of Ireland and Northern Ireland jurisdictions [46]. Regarding study design, six studies were prospective longitudinal [21, 36, 38, 40–42], six were cross-sectional [35, 39, 43, 44, 46, 47], and two were retrospective [37, 45].

**Table 1:** Summary of findings from included studies in the scoping review

Authors (year of publication)	Objective	Design	Setting	Methods	Cancer type and sample size	PROMs used	Interventions	Results
Ashley, L. et al. (2013) [21]	To test the technical and clinical feasibility of innovative electronic Patient-reported Outcomes from Cancer Survivors (ePOCS) system	Prospective longitudinal	Single cancer center in the UK	Patients with cancer, in their first 15 months post-diagnosis, were recruited and consented to complete questionnaires via ePOCS at 3 time points: at 6 (T1), 9 (T2), and 15 (T3) months	Cancer type: Potentially curable breast, colorectal, and prostate Sample size: 636 recruited (consented participants) 597 invited patients to complete the questionnaire at all 3 time-points	The Illness Perception Questionnaire-Revised (IPQ-R), The EuroQol- 5D, version 2 (EQ- 5Dv2), The Medical Outcomes Study 36-Item Short-Form Health Survey, version 2 (SF- 36v2), The Social Difficulties Inventory (SDI- 21), The European Organisation for Research and Treatment of Cancer (EORTC) Quality of Life Questionnaire (QLQ) (EORTC-QLQ), The Quality of Life in Adult Cancer Survivors (QLACS) scale	Recruitment strategy: -Face-to-face: Patients were informed about the study in-person during a routine hospital visit -Letter and telephone: If patients were not informed in-person, they were sent a letter about the study signed by their consultant or were contacted by telephone Follow up: -At each time-point, a maximum of 3 email/letter reminders were sent -The ePOCS system Tracker automatically generates all the administrative actions due each day (i.e., all the patient invitations, reminders, thank you, etc., that need sending on that date) After survey completion: Patients received a thank you communication and a paper feedback questionnaire with a prepaid envelope	Recruitment using different strategies: - Face-to-face in clinic (61.4%, 490/798) - Letter (41.0%, 125/305) - Telephone (48.8%, 21/43) Response rate at different time points (fully/partially completed questionnaires): -At T1: 85.1% (541/636) -At T2: 70.0% (442/631) -At T3: 66.3% (414/624) Response rate across all 3 time points: - Response rate: 57.1% (*n* = 341) - 82 required no reminders (24.0%, 82/341) - 92 required 1 or more reminders at all 3 time-points (27.0%, 92/341) − 167 (49.0%, 167/341) received 1 or more reminders at 1 or 2 time-points Partially completed questionnaire% across all 3 time-points: 31.7% (*n* = 189)
Baker, BG. et al. (2022) [43]	To compare breast-conserving surgery PROs with those of immediate implant-based and free flap reconstruction	Comparative cross-sectional	Single tertiary center in the UK	Patients with cancer who underwent surgery were contacted 6 to 12 months postoperatively	Cancer type: Breast Sample size: 271 contacted patients 190 patients returned completed questionnaires	The “satisfaction with breasts” BREAST-Q™ Breast-Conserving Therapy or reconstruction module as appropriate	Data collection: Patients were contacted by postal mail. They were provided with a cover letter, the questionnaire, and a prepaid envelope enclosed to facilitate the return Non-respondents: Non-respondents were sent a reminder letter and an additional copy of the questionnaire following a 5-week interval period	Response rate before reminder: 57% (*n* = 154) patients returned a completed questionnaire immediately Response rate after reminder: 70% (*n* = 190), 36 additional patients returned questionnaires
Beebe-Dimmer, JL. et al. (2018)	(1) To provide data on the feasibility of collecting epidemiologic and quality of life data using a self-administered, web-based survey (2) To determine the feasibility of using an at-home saliva collection kit for future investigations (3) To provide preliminary data characterizing the target population	Cross-sectional	Academic cancer institute in the USA	-Cancer survivors of less than 5 years -A letter was sent to all eligible patients’ treating physicians asking if there was any reason patient contact was not advised. Patients were then invited to complete the survey	Cancer type: Primary lung, breast (female), prostate, or colorectal Sample size: 1475 patients were sent the recruitment letters	Functional Assessment of Cancer Therapy (FACT) questionnaire	Data collection: Patients were mailed an introductory brochure, for completing the baseline survey online, and a study phone number if they preferred interviewer assistance Non-respondents: - Interviewing staff called participants who did not complete the baseline survey within two weeks - Each participant was contacted with up to nine calls at varying times and days of the week including weekends After survey completion: Participants received a $25 gift card and those who consented were sent a saliva collection kit, a pre-paid return envelope, and a consent form for future follow-up	Overall response rate: 67.8% (*n* = 1000) Online survey response rate: - 30% (*n* = 300) out of 1000 participant completed the baseline survey online - 96% of all questions were answered Interviewer-administered survey response rate: - 70% (*n* = 700) of participants required an interviewer-administered survey by telephone - 97% of all questions were answered
Brønserud, MM. et al. (2019) [40]	To examine the feasibility of a nationwide collection of PROs in a population with lung cancer and to describe the characteristics of respondents compared to non-respondents	Prospective longitudinal	17 hospital departments treating patients with lung cancer in Denmark	-Cancer patients were identified from the Danish Lung Cancer Registry between 2013 and 2015 -PROs were administered by two different modes (Project part 1 vs part 2) -Patients received the questionnaires before treatment at baseline (“QoL0”), 3 months (“QoL1”), 6 months (“QoL2”), and 12 months (“QoL3”) after the date of first treatment	Cancer type: Lung Sample size: 7295 eligible patients 4229 patients completed at least 1 questionnaire	Danish versions of the European Organisation for Research and Treatment of Cancer (EORTC) QLQ-C30 (“Quality of Life Questionnaire Core 30”) and EORTC QLQ-LC13 (“Quality of Life Questionnaire Lung Cancer 13”)	Project part 1: -QoL0 handed out as paper questionnaire at the first visit to the outpatient clinic -QoL 1–4 were sent out as paper versions by mail -Patients were also given the option to complete the questionnaire electronically -Letter reminders were sent to non-respondents after 14 days, and a new questionnaire after 42 days Project part 2: -Patients were informed about the project at baseline and were asked to fill out the questionnaire online -Electronic versions of the questionnaires were delivered and filled out using either computer at home or on a tablet in the waiting room, in association with planned hospital visits for follow-up and treatment -Patients were reminded by the department staff to fill out questionnaires at each visit	Overall response rate: In total, 4229 patients with lung cancer (58%) completed at least 1 questionnaire within the chosen time windows Project part 1, response rate at each time point: At QoL0: 10.1% (*n* = 507) At QoL1: 40.6% (*n* = 1732) At QoL2: 57.8% (*n* = 2143) At QoL3: 49.0% (*n* = 1405) Completed questionnaires in total: *n* = 5787 Project part 2, response rate at each time point: At QoL0: 2.4% (*n* = 55) At QoL1: 49.9% (*n* = 924) At QoL2: 56.3% (*n* = 857) At QoL3: 48.7% (*n* = 503) Completed questionnaires in total: *n* = 2339
Drummond, FJ. et al. (2015) [46]	To establish an international PROs study among prostate cancer in two countries with different healthcare systems and ethical frameworks	Cross-sectional	Two population-based cancer registries in Republic of Ireland (RoI) and Northern Ireland (NI) jurisdictions	-Potential participants (survivors up to 18 years post-diagnosis) were identified from cancer registries and assessed for eligibility by health care professionals - Survivors were invited to complete a postal survey at one time point -Participants were survivors, grouped depending on number of years post-diagnosis: short-term (< 5 years), long-term (5–9.9 years) and very long-term (≥ 10 years)	Cancer type: Prostate Sample size: 22,823 identified survivors 12,322 country-stratified random sample 6559 eligible survivors 6262 survivors who received the survey	A core questionnaire was developed: General and prostate cancer-specific HRQoL were measured using EORTC QLQ-C30 and QLQ-PR25 instruments. The EuroQoL EQ- 5D- 5L to measure utility. The 21-question Depression, Anxiety and Stress Scale (DASS- 21) to evaluate psychological well-being. Treatment decision-making and decisional regret measured using the Control Preference Scale, and Decision Regret Scale (DRS)	Methods to maximize survey response: -Dillman’s Tailored Design Method and methods that previously demonstrated an increase in response were used - Phone number(s), one in each jurisdiction, with instructions on paper questionnaires and information leaflets, were provided when needed - Men in RoI were randomized to receive either conditional or unconditional modest financial incentives. No incentives were used in NI -The study was branded to participants as the PiCTure (Prostate Cancer Treatment, your experience) study	Response rate: - Overall response rate: 53.5% (3348/6262) - RoI jurisdiction response rate: 53% (2338/4453) - NI jurisdiction response rate: 48% (1010/2106) - 54% (*n* = 1614) of the respondents were short term survivors - 53% (*n* = 1734) of the respondents were long term survivors (*n* = 1075) and very long-term survivors (*n* = 659) Freephone use: - 14% (*n* = 906/6559) of all questionnaire recipients or their families called the freephone for different reasons - Only 7%; (60/906) completed the questionnaire by telephone
Horevoorts, NJ. et al. (2015) [44]	To study the influence of two methods of invitation on the response rate and to examine the preference of questionnaire mode of administration (paper or web-based) for the older patient in particular	Cross-sectional	Large population-based PROs registry in the Netherlands	Patients (2–3 years since diagnosis) were divided into 2 groups for this study: -Paper-optional group -Paper-included group	Cancer type: Colorectal Sample size: 3406 invited patients in the paper-optional group 179 invited patients in the paper-included group	The Self-Reported Comorbidity Questionnaire, Problem Areas in Diabetes Questionnaire, EORTC QLQ-C30, EORTC QLQ-CR38, SF- 12, DS14, Brief Illness Perception Questionnaire, Fatigue Assessment Scale, Hospital Anxiety and Depression Scale	The paper-optional group: -Patients were invited via a letter from their former attending specialist -The letter contained a website address and log-in instructions to fill out the web-based questionnaire - It also contained a reply card with a prepaid return envelope so participants could request a paper version of the questionnaire The paper-included group: - Patients received the same letter but with a paper questionnaire and a prepaid return envelope - They could also choose to fill out the web-based questionnaire with the included access code Non-respondents from both groups: A reminder letter with a paper questionnaire and a prepaid return envelope was sent to non-respondents from both groups after an average of 3 months	The paper-optional group response rate: - 73.14% (*n* = 2491) of the 3406 invited patients completed the questionnaire - For respondents aged 70 years and older the response rate was 68.84% (1290/1847) - 71% of those 70 years of age and older used paper questionnaires - Online response rate was 41.23% (1027/2491) - Among those who responded online, their response rate after the first invitation was 95.42% (979/1027), which was significantly higher than the paper respondents (52.36%, 764/1464) The paper-included group response rate: - 74.9% (*n* = 134) of the 179 invited patients completed the questionnaire - For respondents aged 70 years and older, the response rate was 75.7% (78/103) - Online response rate was 12.7% (17/134) Reminders: Sending a reminder increased the response by 30% in both arms
Juul, T. et al. (2021) [41]	To test the feasibility of a new method for systematic screening for late sequelae following colorectal cancer treatment	Prospective longitudinal	Surgical departments at 5 hospitals in Denmark	- Patients with cancer who underwent surgery within the past 3 years before the study started -PROs were administered by two different modes -Patients were initially sent an invitation letter to the study -Survey was sent to those who responded to the invitation letter at 3, 12, 24, and 36 months after surgery as part of follow-up	Cancer type: Colorectal Sample size: 1721 invited patients 1386 included patients (responded to the initial invitation)	Different questions from multiple instruments were incorporated into one survey, in addition to ad hoc items—see original article for list of instruments—Low Anterior Resection Syndrome Score, Wexner Incontinence Score, St. Mark’s Incontinence Score, Bristol Stool Chart, Patient Assessment of Constipation Symptoms, Colostomy impact score, Memorial Sloan Kettering Cancer Center Bowel Function Index (dietary scale), ICIQ Female Lower Urinary Tract Symptoms, ICIQ Male Lower Urinary Tract Symptoms, Rectal Cancer Pain Score, International Index for Erectile Function, Rectal Cancer Female Sexuality Score (sexually active only), European Quality of Life—5 Dimensions (EQ5D)	Electronic survey: An invitation with a link to the survey was sent to a secure digital mailbox (eBoks) linked to Danish citizens’ civil registration number Paper survey: Mailed to non-users of the internet, along with a prepaid return envelope Non-respondents to the invitation letter (including patients having completed at least 1 survey but not responding to the subsequent one): Sent two reminders, 2 weeks apart	Overall response rate: A total of 1365 (79.3%) completed at least one survey (551 completed the survey once, 637 twice, 172 thrice, and 5 completed the survey 4 times) Participants in the electronic mode of administration%: 72.5% (*n* = 989) Participants in the paper mode of administration%: 27.6% (*n* = 376) Participants after the initial invitation to the study%: 73.4% (*n* = 1018) Participants included after one reminder%: 15.9% (*n* = 220) Participants included after two reminders%: 10.7% (*n* = 48)
Kim, SB. et al. (2012) [36]	To assess the feasibility of two patient-reported health-related quality of life (HRQOL) instruments, and determine which domains are most sensitive to HRQOL outcome after renal surgery	Prospective longitudinal	Urology surgical department at a university-affiliated hospital in the USA	- Patients received surveys at preoperative (baseline) visit and at 2, 4, 12, and 24 weeks after surgery - Patients were given the choice of two different versions of the instrument	Cancer type: Renal Sample size: 82 eligible patients 60 patients included in the final cohort	Convalescence and Recovery Evaluation (CARE), and SF- 12	Modes of administration: Patients were given the choice of an internet or paper version of the instruments Non-respondents: Patients were contacted via phone or email on up to 3 separate occasions if the survey was not complete 1 week after due date	Patients who completed the baseline questionnaires%: 86.6% (*n* = 71) out of 82 Patients completed at least one CARE and SF- 12 questionnaires during the perioperative period%: 85% (*n* = 60) Completed online surveys%: 51% Completed paper surveys%: 49%
Millar, MM. et al. (2019) [47]	To compare and assess response to a PROs web survey to that of a paper survey among individuals with cancer via a central cancer registry and to assess the effect of including an informative brochure describing the cancer registry on response outcomes	Cross-sectional	Central cancer registry in the USA	Sample consisted of two groups of cancer survivors: -The first group were those diagnosed within 12 months -The second group were longer-term survivors (≥ 5 years post-diagnosis for those with colorectal, breast, and prostate cancer, and ≥ 3 years for ovarian cancer and multiple myeloma) -Individuals were pooled and randomly assigned to receive the questionnaire via two methods - A brochure describing the role of a central cancer registry and its involvement with research activities was designed and included similar imagery from the questionnaire	Cancer type: Colorectal, breast (female only), prostate, ovarian, and multiple myeloma Sample size: 446 eligible individuals	The questionnaire was newly developed for this study. It consisted of up to 35 items including questions on current health, cancer recurrence, and willingness to participate in various kinds of cancer research	Questionnaire mode: -Individuals were randomly assigned 1:1 to receive either a paper or web questionnaire -The paper survey arm: All communications mentioned response by paper questionnaire only -The web arm: All communications mentioned response via the web-based questionnaire only Brochures: Within each survey mode experimental arm, individuals were then randomly assigned 1:1 to either receive a brochure or not receive a brochure in the first mailing Questionnaire administration: -For both survey modes, the initial mode of contact was postal mail - Potential participants received up to four mailings (pre-notification letter with or without brochure, invitation packet with either questionnaire and stamped return envelope or web survey instructions for the web arm, thank-you/reminder letter, and a second replacement packet) -Individuals identified as Hispanic were sent bilingual English and Spanish invitation letters, and the questionnaires for both arms were available in Spanish upon request Non-respondents: -In addition to the reminder letters and the replacement packet, telephone calls were made as the last stage of the recruitment protocol -Up to three calls were made at varying times of the week to encourage each individual to respond via the mode they were assigned, also the option of responding via telephone was offered	Overall response rate: - 46.9% (209/446) completed the survey across all study arms combined - 25.4% of responses were obtained after the first invitation packet Reminders and subsequent contact: - 31.1% of the responses were after the reminder letter - 23.9% after the second invitation packet - 19.6% after the final phone call stage Response rate in paper survey mode: 50.4% (114/226) Response rate in web survey mode: 43.2% (95/220) Response rate by brochure assignment: Brochure sent: 48% (106/221) Brochure not sent: 45.8% (103/225) Response rate by brochure assignment and survey mode: Paper, no brochure: 50.4% (58/115) Paper, brochure: 50.5% (56/111) Web, no brochure: 40.9% (45/110) Web, brochure: 45.5% (50/110)
Nelson, JA. et al. (2022)	To describe a quality improvement initiative to improve patient engagement with the BREAST-Q for breast reconstruction	Retrospective	Academic cancer center in the US	2011–2017: Electronic questionnaires were assigned at time points relative to a patient’s initial surgery date (e.g., pre-operative, 2-week post-operative, 1 month post-operative) 2018–2019 (quality improvement initiative to increase completion rates): Electronic questionnaires were administered at every clinic visit	Cancer type: Breast Sample size: 38,582 BREAST-Q questionnaires	BREAST-Q	2011–2017: - Patients received email reminders only whenever a questionnaire time point approached - Questionnaires were not available pre-visit, so they were not part of discussions with patients 2018–2019: - Questionnaires were linked to clinical visits to both patients and clinical staff and included as part of discussions with patients - In-house electronic PROM platform was used to assign patients the BREAST-Q within 1 month prior to their visit - Non-respondents were asked to complete the questionnaire in clinic on a tablet - Each clinical site was appointed a “BREAST-Q Champion,” responsible for managing BREAST-Q-related matters - Implemented real-time monitoring of BREAST-Q completion rates	2011–2017: (22,730 questionnaires were collected) - 61.4% of all BREAST-Qs collected from 2011 to 2019 were completed in this 7-year period - Annual response rates ranged from 36.7 to 48.2%, with an overall response rate of 42.8% over the 7-year period 2018–2019: (15,852 questionnaires were collected) - 38.6% of all BREAST-Qs were collected in this 2-year period after the intervention - Annual response rates were 63.3% in 2018 and 87.6% in 2019, with an overall response rate of 76.4% over the 2-year period
Nielsen, LK. et al. (2019)	To investigate whether electronic reminders and real-time monitoring improve PROs completion rate	Prospective longitudinal	All 10-hematology departments in multiple national centers in Denmark	-Newly diagnosed or relapsed patients, who required treatment, completed questionnaires at study entry and at 12 follow-up time points during a 24-month period -The follow-up target dates were every 4 weeks for the first 6 months and thereafter every 3 months until 24 months - “On-time responses”: Questionnaires completed before or within the 7-day response window - “Salvage response”: Questionnaire completed at day 7 after the target date or later -The remainder were categorized as a “never response”	Cancer type: Multiple myeloma Sample size: 481 eligible and invited patients 271 patients reached the first follow-up PROs assessment	European Organisation for Research and Treatment of Cancer Quality of life QLQ-C30 (EORTC QLQ-C30), the Multiple Myeloma module QLQ-MY20 (EORTC QLQ-MY20), the Chemotherapy-Induced Peripheral Neuropathy module (EORTC QLQ-CIPN20) and the Short-form health survey version 2 (SF12v2)	Real-time monitoring: -All patients were informed about the importance of completing the questionnaires as a baseline inclusion interview for the study -All local nurses were educated and trained on the importance of completing the questionnaires and their role in minimizing the missing data during follow up -Departments were informed about their role in targeting non-responders and were financially compensated Web-based questionnaire: A link was automatically sent to the patients’ email on the target date Paper-and-pencil questionnaires: -Patients had the option to this method if preferred -Questionnaires were given to patients at two time points as three sets of questionnaires each scheduled appointment with the target date for completion written on the cover page - Patients were handed the three sets of questionnaires for a total of three times by the local nurse At baseline: Patients completed the questionnaires alone or with assistance from the study nurse using a tablet or on paper Follow-up: -The study nurse confirmed that the questionnaires were completed by the day of treatment, they also provided support in questionnaires completion when needed Non-respondents: -Those who were using the web-based questionnaires received automatic reminders emails - For patients who didn’t complete the questionnaire on day 7 after the target date, the local study nurse contacted them within 2 weeks, documented the reason for non-response and invited them to complete the questionnaire	- For the study cohort (*n* = 271), 1441 scheduled follow-up questionnaires were expected to be completed at the time of analysis Electronic completion: Chosen by 85% out of 271 The paper-and-pencil method: Chosen by 15% Questionnaires completed on-time%: 84% (*n* = 1214 out of 1441) represent the completion rate Questionnaires not completed on-time% (*n* = 227 out of 1441): 153 (67%) were “salvage responses” and 74 (33%) were “never response” Reminders: - A significantly higher proportion of uncompleted questionnaires were completed after the patients had received the electronic reminder (*p* < 0.001) - At day 4: 25.5% (*n* = 100) out of 392 not completed electronic questionnaires were completed - At day 3: 16.1% (*n* = 75) out of 467 not completed questionnaires were completed Real-time monitoring effect: - A significantly higher proportion of uncompleted questionnaires was completed after real-time monitoring (*p* < 0.001) - At day 6: 15.7% (*n* = 35) out of 223 not completed questionnaires were completed -At day 7: 30.7% (*n* = 58) out of 189 not completed questionnaires were completed
Van den Berg, L. et al. (2021)	To evaluate the feasibility and accuracy of daily micro-surveys using a smartphone platform among patients recovering from cancer surgery	Prospective longitudinal	Urban, tertiary-care hospital and cancer center in the USA	- The full-length SF- 36 was administered preoperatively at the time of enrollment and 4, 12, and 24 weeks postoperatively - By the fourth day after enrollment, patients were asked to complete daily “micro-surveys” for the entire study period	Cancer type: Breast, primary melanoma, sarcoma, thyroid, parathyroid, primary gastric, colorectal, primary or metastatic hepatic and peritoneal lesions Sample size: 95 patients consented and downloaded the application	The RAND Short Form- 36 (SF- 36, version 1.0)	Smartphone application: Patients downloaded an application that offered both the full-length of the survey and the daily micro-surveys comprising five randomly selected items from the Short Form- 36 (SF- 36) - Micro-surveys were administered without replacement until the entire SF- 36 was administered weekly	Overall mean response rate: - Response rate to the intermittent full-length SF- 36 was76% - Response rate to the micro-surveys was 34% Patients who responded to surveys in the preoperative time interval%: 55% (*n* = 52) Patients who responded to surveys in the first 6 weeks postoperative interval%: 78% (*n* = 74) Patients who responded to surveys 6 + weeks in the postoperative interval%: 33% (*n* = 62) Patients who did not respond to any surveys%: 4% (*n* = 4)
Van Egdom, LSE. et al. (2019) [45]	To describe a step-by-step overview of the framework deployed for implementation of both provider and PROs data and discusses the challenges within the implementation process	Retrospective	Single academic breast cancer center in the Netherlands	Time-points for data collection: baseline (prior to treatment; T0), following the last course of neoadjuvant systemic therapy (T3), 6 months after surgery (T6) and annually thereafter (T12 - 60) Data collection: Different pathways were constructed according to patients’ treatment plan since the used BREAST-Q questionnaire was surgery-specific (i.e., breast Conserving Therapy, mastectomy alone, and mastectomy with breast reconstruction, either with implants or autologous)	Cancer type: Breast Sample size: 239 enrolled patients	The EORTC-QLQ-Core (C30), EORTC-QLQ Breast Cancer (B23), BREAST-Q (both preoperative and postoperative modules), EQ- 5D- 5L, Distress Thermometer, the Reproductive Concerns Scale (RCS-NL), and the CarerQoL- 7D	Data collection tool: An in-house electronic data collection tool was developed which allowed the construction of data collection-forms and automatic distribution of PROMs - Emails were sent to the patients to activate the distribution of PROMs Automatic PROMs: After the right treatment pathway was selected by the physician, all the following PROMs were sent automatically by the tool at the right time-point -The tool was linked to the electronic health record to allow physicians discussion of the data at the patients’ clinic visit Non-respondents were approached by the specialist nurse to document their reason for not completing the questionnaire	T0 response rate: 83.3% (199/239 patients) T6 response rate: 65.7% (157/239 patients) T12 response rate: 55.1% (108/196 patients)
Bulkley, JE. et al. (2019)	To evaluate the impact of repeated contacts on PROs response rates and missing data after surgery in patients with cancer	Cross-sectional	Research center in the USA	- Patients with advanced bladder cancer that had undergone complete cystectomy with either an ileal conduit or neobladder UD were included -Patients were recruited 6 months postoperatively	Cancer type: Bladder Sample size: 379 patients were sent a survey	Different questions from multiple instruments were incorporated into one survey: Quality of life measures EQ- 5D (5 items), Body image scale (BIS) (10 items), Bladder Cancer Index (BCI) (20 items), City of Hope Quality of Life (COH-QOL) (42 items), Current sexual activity (1 item), Shared decision-making (4 items), Goal dissonance (10 items), Decision regret (5 items), Informed decision-making questions designed for this study (4 items), Healthcare expenses questions designed for this study (15 items)	Survey administration: -Postal mail was used to send surveys along with a cover letter and an information sheet explaining the study -A $25 incentive for participants was described in the mailing packet Follow up: - Telephone calls were made beginning 10 days after the mailing and occurred every 2–7 days - Calls continued until there was a survey return or a decline to participate or until a maximum of 10 calls were made - Patients were offered to complete the survey via telephone or have a new mailing packet sent - Missing data were reviewed, and participants were contacted to complete those items by telephone	Response rate: 71% (269/378) returned completed surveys Follow-up outcome: -First contact (receipt of survey) resulted in 27.4% (104/379) response rate -Second contact (first phone call) resulted in 22% response rate -Third contact (second phone call) resulted in 20.5% response rate -Subsequent follow-up contact response rate fell gradually until attenuation at about 10% by contact 6

### Studied population demographics and quality assessment

The demographics of studied populations are outlined in Table 2. Five studies (35.7%) included a population in which the majority were females compared to five studies (35.7%) where the majority were males, while three studies (23.1%) included all females, and one (7.7%) included all male samples. Only six studies reported race, in which five studies (35.7%) included a majority of White populations, and one study reported a majority of African American population (7.7%). In terms of quality rating, four studies (28.6%) were labeled as “Fair,” and 10 studies (71.4%) were labeled as “Good,” while no studies were labeled as “Poor.”

**Table 2:** Study quality and population demographics

Authors	Age in years	Sex *n* (%)	Race/ethnicity *n* (%)	Quality rating^a^
Ashley, L. et al. *	Mean 61.3 (SD ± 11.09)	Female: 362 (56.9) Male: 274 (43.1)	White British: 523 (97.0) White others: 10 (1.9) British minority ethnic group: 6 (1.1)	Good
Baker, BG. et al. **	Mean 60 (SD ± 11)	Female: 190 (100)	NR	Good
Beebe-Dimmer, JL. et al.*	Median 60 (range 27–79)	Female: 618 (61.8) Male: 382 (38.2)	Non-Hispanic White: 414 (41.4) African American: 586 (58.6)	Good
Brønserud, MM. et al.**	Mean 69, range (17–96)	Female: 3659 (50.2) Male: 3636 (49.8)	NR	Good
Drummond, FJ. et al.**	≤ 59: *n* = 1451 (62%); 60–69: *n* = 3058 (58%); ≥ 70: *n* = 2129 (46%)	Male: 6559 (100)	NR	Good
Horevoorts, NJ. et al.*	Mean 69.41 (SD ± 9.53)	Female: 1177 (44.84) Male: 1448 (55.16)	NR	Good
Juul, T. et al.**	≤ 65: *n* = 470 (27.3%); 65–75: *n* = 625 (36.3%); ≥ 75: *n* = 626 (36.4%)	Female: 785 (45.6) Male: 936 (54.4)	NR	Good
Kim, SB. et al.*	Age, median (IQR): Partial nephrectomy: 59 (44, 67) Radical nephrectomy: 58 (50, 67)	Partial nephrectomy: Female: 9 (25) Male: 27 (75) Radical nephrectomy: Female: 9 (26) Male: 26 (74)	NR	Good
Millar, MM. et al.**	< 40: *n* = 102 (22.9%) 40–49: *n* = 212 (47.5%) 50–59: *n* = 98 (22.0%) ≥ 60: *n* = 34 (7.6%)	Female: 252 (56.5) Male: 194 (43.5)	White: 416 (93.3) Nonwhite: 30 (6.7) Hispanic: 68 (15.5) Non-Hispanic: 372 (84.5)	Fair
Nelson, JA. et al.**	Average 50.0 (SD ± 10.2)	Female: 7119 (100)	White: 5444 (76.5) Black: 539 (7.6) Hispanic or Latino, any race: 443 (6.2) Asian/Pacific Islander: 423 (5.9) Native American/Alaskan Native: 2 (0.03) Other/unknown: 268 (3.8)	Fair
Nielsen, LK. et al.**	Newly diagnosed patients: Mean 67.0 (SD ± 9.7) Relapse patients: Mean 68.7 (SD ± 9.5)	Newly diagnosed patients Female: 70 (42) Male: 95 (58) Relapse patients: Female: 42 (33) Male: 85 (67)	NR	Good
Van den Berg, L. et al.*	Mean 52.1 (SD ± 12.9)	Female: 63 (66.3) Male: 32 (33.7)	Non-Hispanic White: 83 (88) Non-Hispanic Black: 3 (3) Hispanic: 3 (3) Asian: 2 (2) Other: 2 (2) Unknown: 2 (2)	Good
Van Egdom, LSE. et al.*	NR	Female: 239 (100)	NR	Fair
Bulkley, JE. et al.*	Age < 70 years 119 (44%) Age ≥ 70 years 150 (56%)	Female: 57 (21) Male: 212 (79)	Non-Hispanic White: 210 (78) Other race–ethnicity: 59 (22)	Fair

*NR* not reported

^*^Numbers are descriptions of the survey respondents

^**^Numbers are descriptions of all eligible patients who were initially contacted

^a^Study quality was assessed according to the National Heart, Lung, and Blood Institute Study Quality Assessment Tools

### PROs mode of administration

PROs mode of administration varied between electronic only (four studies, 28.6%) [21, 37, 38, 45], paper only (two studies, 14.3%) [39, 43], electronic and paper (six studies, 42.9%) [36, 40–42, 44, 47], and electronic and telephone (two studies, 14.3%) [35, 46] (Table 1). Ashley et al. [21] reported a response rate ranging from 65 to 85% at different time points of fully or partially completed questionnaires when electronic PROs were the only available method. Similarly, Juul et al. [41] found that among a sample of 1365 participants, 72.5% responded electronically compared to 27.6% who used the paper mode sent out to target those without internet access. Nielsen et al. [42] reported providing both web-based and paper-and-pencil questionnaires, where 85% chose the electronic method to complete at baseline and at 12 follow-up time points, whereas Kim et al. [36] and Brønserud et al. [40] reported similar overall response rates between paper and electronic PROs modalities with a minimal difference ranging from 1 to 2%. Horevoorts et al. [44] reported that respondents aged 70 years and older chose to fill out a paper questionnaire more often (71.0%, 677/954) compared to electronic. Telephone-administered surveys were used by a study resulting in completion rates ranging from 7 [46] to 70% [35].

### Interventions implemented to improve PRO response rates

Numerous interventions were implemented in each study methodology resulting in varying influence on the PROs response rates (Table 1). Reminder systems were used in 12 (85.7%) studies [21, 35–37, 39–45, 47] differing in the frequency and mode (e-mail, postal mail, telephone, and in-person). However, only seven (50%) studies [21, 39, 41–44, 47] reported the observed changes in the response rate after implementing reminders as an intervention. Recruitment strategies, defined as measures taken at the beginning of the study to raise patient awareness of PROs to encourage completion, were clearly described throughout five studies (35.7%) [21, 35, 43, 44, 47]. These strategies were used during a duration ranging from 6 months up to 3 years post-diagnosis across the studies. They included face-to-face interactions, telephone, informative brochure, and cover letter or a letter from former attending. Dillman’s Tailored Design methods [48] were employed to increase response rate in one study (7.1%) [46]. Finally, two studies (14.3%) [37, 42] reported the influence of real-time monitoring which included clinic staff engagement, patient-provider PROs scores discussion, or assigning a PROs champion to each clinic among other interventions.

### Type of intervention: Reminder systems

Baker et al. [43] employed reminders in the form of mailed letters, along with an additional copy of the paper questionnaire and a prepaid return envelope, to patients following a 5-week interval period from the initial contact. They reported that 36 additional patients returned a completed questionnaire resulting in an increase in the completion rate from 57 to 70%. Millar et al. [47] employed two methods to deliver reminders: postal mail letters sent twice after the first mailed invitation, and phone calls made up to three times during the final stage. Postal mail reminder letters yielded 31.1% of the total study responses, and 23.9% of the responses were obtained after a second invitation packet was mailed, while 19.6% were completed after the final telephone stage. Likewise, Nielsen et al. [42] reported that a significantly higher proportion of uncompleted questionnaires was completed after patients received an automated electronic reminder (*p* < 0.001), while Horevoorts et al. [44] reported a 30% improvement in the total response rate after sending a reminder letter with a paper questionnaire and a prepaid return envelope. The impact of repeated telephone reminders on PROM response rates and missing data was evaluated by Bulkley et al. [39]; they reported an overall response rate of 71%, in which they received of 27.4% completed surveys after the initial postal mail in addition to 22% and 20.5% completed surveys after first and second telephone reminder calls. Juul et al. [41] showed that out of 1365 participants who completed the questionnaire, 15.9% responded after receiving one reminder and 10.7% after receiving two. The efficacy of reminders in increasing patient responses was also demonstrated in another study [21] reporting that over 70% (259/341) of the respondents required a reminder to return a fully completed questionnaire, in which 49% (*n* = 167) required one or more reminders at one to two time points and 27% (*n* = 92) required one or more reminders at three time points.

### Type of intervention: Real-time monitoring and clinic staff engagement

Real-time monitoring strategy included informing all patients about the importance of completing the questionnaires, training all local nurses on minimizing the missing completion during follow up, and notifying departments about their role in targeting non-responders. Additionally, they included financial compensation for participating departments. Nielsen et al. [42] investigated the role of real-time monitoring to improve completion rates. Their results show that a significantly higher proportion of uncompleted questionnaires was completed after real-time monitoring (*p* < 0.001), in which 15.7% and 30% of the noncompleted questionnaires were completed at days six and seven of the follow up, respectively. Clinic staff engagement was also assessed by Nelson et al. [37] who described operational interventions as part of an organizational level quality improvement initiative to encourage patients with breast cancer to interact with PROs. They reported an overall completion rate of 76.4% over the 2-year period of the quality improvement compared to a previous 42.8% completion rate.

### Type of intervention: Patient education and recruitment

Ashley et al. [21] used three recruitment strategies in which patients were approached and given further details about PROs. They found that the most effective recruitment strategy was face-to-face during a regular clinic visit (61.4%, 490/798) compared with letter (41.0%, 125/305) and telephone (48.8%, 21/43). Millar et al. [47] evaluated the efficacy of employing an informative brochure which included an imagery from the questionnaire sent by postal mail along with a pre-notification letter. The proportion of respondents was not significantly affected by the inclusion of a brochure in the recruitment materials; 48.0% of those allocated to the brochure intervention responded, compared to 45.8% of those who were not. Other studies described employing similar recruitment strategies; however, their influence on the response rates was not reported. Beebe-Dimmer et al. [35] included an introductory brochure and a $25 gift card sent after survey completion; Baker et al. [43] included a cover letter; Bulkley et al. [39] used a mailing package which included a cover letter, an information sheet, and a notification of a $25 incentive for participants; and Horevoorts et al. [44] invited patients via a letter from their former attending specialist. A study by Drummond et al. [46], who reported an overall response rate of 53%, used pre-established methods to maximize response rate, including Dillman’s Tailored Design methods [48]. They reported a completion rate of 53% in a jurisdiction in which the population was randomized to receive either conditional or unconditional modest financial incentives, and a completion rate of 48% in another jurisdiction in which no participant received any incentive.

## Discussion

For PROMs to be acted upon, patients must make the effort to complete them, so the benefits of PROs implementation are fully realized. Yet, PROMs completion rates remain low in real life settings. This review examined the existing literature to synthesize various interventions that had been implemented to increase PROM completion in real-life or non-clinical trial research settings among patients with cancer. This could be an important step towards deepening the current understanding of the needed strategies and operational changes to enhance PROs adoption and completion by patients. To the best of our knowledge, this is the first scoping review that explores current real-life and non-clinical trial PROM completion strategies. We found that several interventions were implemented to maximize PROM completion, each yielding a different influence on completion rate. They included reminder systems, increasing clinic staff engagement, patient education, and incentives. This study specifically reported different settings, cancer types, patients’ demographics, PROM instruments, and finally, study quality rating of all included articles.

PROs implementation beyond clinical trials settings into clinical practice as part of routine patient care has its own challenges [25, 26]. Capturing and utilizing PROs can be influenced by logistical barriers, including added staff workload, workflow, data collection strategies, selection of suitable PROMs, management of reported symptoms, and cost [27–31]. Furthermore, patients’ compliance, health literacy, technological skills, language barriers, and enthusiasm towards PROs could significantly limit overall completion [25, 31, 32]. If not addressed, these barriers could limit the benefits of PROs and widen the health disparities that currently exist among minoritized individuals.

PROMs completion varied within studies depending on modes of delivery whether electronic, paper, or other methods. While previous research discussed the potential barriers of using electronic PROs including sample bias, technology illiteracy, lack of internet, and smart device access [49], some studies reported a completion rate above 60% among diverse demographics using electronic PROs. Considering that electronic PROs had high response rates, a question could be raised hypothesizing that providing patients with web-based questionnaires solely may result in higher response rates, faster data availability, and a more sustainable approach of integrating PROs into everyday workflow. However, some studies reported participant’s preference for paper questionnaires; thus, this may support having paper questionnaires available in addition to electronic versions. It should be noted that there are disadvantages to using a model in which there is complete dependence on department staff to inform patients about questionnaires. This might emphasize the necessity of using an automated design to deliver questionnaires at assigned time points to alleviate staff burden and ensure higher patient inclusion.

PROs questionnaires are typically assigned at time points that mark an important stage in the patient’s evaluation, i.e., at baseline, pre-provider visit, during treatment, at hospital admissions, and post-treatment. Having numerous completed PROM questionnaires available across the care timeline might facilitate capturing longitudinal changes in the patient’s health. Therefore, patients’ lack of compliance to PROM completion may influence whether PROs benefits are denied to non-completers. Factors contributing to non-completion could be related to modifiable circumstances, such as participants forgetting about the questionnaire in the assigned timeframe. Thus, implementation of reminders could be one of the main interventions used to reduce non-response and improve completion compliance over time. Another intervention could be the used of clinical staff to assign PROMs to patients during clinic visits and possibly assist them with questions they may have. Previous studies identified that one of the patient-level barriers to PROM completion was the lack of adequate explanation regarding the objective of PROs and the value of collecting these data; therefore, educating patients about the benefits and how PROMs are used by their providers to improve their health could lead to increased completion rates [50–52]. Finally, providing feedback either through providers discussing PROMs results with patients or through dashboards could increase patients’ completion of PROMs.

It should be noted that although some of the strategies described such as clinical staff engagement might work in research settings with a set timeline, their efficacy in influencing PROM completion rate might be difficult to sustain in real-world settings. But others such as improving PROM completion through providing feedback through dashboards may be sustainable. Future efforts may examine measures aimed at improving clinician-patient PRO engagement, specifically by enhancing communication between health professionals and patients, providing feedback to patients on their most recent PROM scores and trends, and emphasizing the importance of explaining the purpose of PROMs as an investment in the patient’s health.

### Limitations

Our scoping review has some identified limitations. First, variation in the literature indexing of appropriate search terms may have limited the comprehensiveness of our database search. To overcome this limitation, a research librarian was enlisted to perform the search. Second, unpublished studies and reports were excluded, as well as studies in languages other than English. This may have omitted data from studies with negative findings or different completion interventions. In addition, this may have excluded some populations that may be underrepresented, limiting the generalizability of the findings. Third, we only included studies that implemented PROs in real-world settings and reported the results of their intervention on completion rate. There is a possibility that some clinical PRO studies had implemented different effective interventions that were not captured in this review. Finally, there is heterogeneity among studies with respect to the used completion interventions, PROMs, and timepoints, which makes comparison across studies difficult.

## Conclusion

This review provides a toolbox for those seeking to incorporate PROMs in non-clinical trial settings. It highlights the wide variety of strategies in PROMs implementation and creates a framework for understanding their impact on completion rates. Reminder systems including paper, text, telephone, or in-person, patient education on benefits of PROs, and clinical staff involvement were shown to be effective in increasing the overall completion rate. Results also emphasize the importance of considering local contexts to identify the most effective interventions for individual implementations of PROMs. Finally, this study underlines the existing gaps where published data remains insufficient, and further research would be advantageous for a more patient-centered, interactive, and sustainable design. Consequently, further work is needed to tackle patient barriers, address logistical issues, and build a more equitable process to help enhance PROs incorporation into routine care.

## Supplementary Information

Below is the link to the electronic supplementary material.

Supplementary file1 (DOCX 15 KB)

## Data Availability

No datasets were generated or analysed during the current study.
