# Supplementary material for: Interventions influencing patient-reported outcomes (PROs) response rates in cancer: a scoping review
Source: J Cancer Surviv. 2025 Apr 15;20(Suppl 1):1058–76. doi: 10.1007/s11764-025-01801-9 (PMC13676535; doi:10.1007/s11764-025-01801-9)
Supplement: Supplementary file 1 [file 11764_2025_1801_MOESM1_ESM.docx]

**Title: Interventions Influencing Patient-Reported Outcomes (PROs) Response Rates in Cancer: A Scoping Review**

**Journal name: Journal of Cancer Survivorship**

**Authors:** *Nada Al-Antary, MD^1^; Samantha Tam, MD, MPH^1,2,3^; Suma Alzouhayli^4^; Theresa Zatirka, MPH^5^; Michael Ryan, MBA, PsyD^6^; Steven S Chang, MD^1,2,3^; Benjamin Movsas, MD^7^; Eric Adjei Boakye, PhD^1,2,3,8^*

^1^Department of Public Health Sciences, Henry Ford Health, Detroit, MI

^2^Department of Otolaryngology – Head and Neck Surgery, Henry Ford Health, Detroit, MI

^3^Henry Ford Health + Michigan State University Health Sciences, Detroit, MI

^4^Wayne State University School of Medicine, Detroit, MI

^5^ Clinical and Quality Transformation, Transformation Consulting, Henry Ford Health, Detroit, MI

^6^Department of Supportive Oncology, Henry Ford Health, Detroit, MI

^7^Department of Radiation Oncology, Henry Ford Hospital, Detroit, MI

^8^Department of Epidemiology and Biostatistics, Michigan State University College of Human Medicine, East Lansing, MI

**Corresponding author**

Eric Adjei Boakye, PhD

Henry Ford Health

Department of Public Health Sciences

One Ford Place

Detroit, MI 48202

Phone: (313) 874-6218

Email: [eadjei1@hfhs.org](mailto:eadjeiboakye49@siumed.edu)

Supplemental Table (1): Search string used to conduct the literature review using the PubMed database

**Different terms were used for each keyword, “AND”/“OR” operators were used to connect the terms, and results were combined to produce the following search string:**

(("cancer s"[All Fields] OR "cancerated"[All Fields] OR "canceration"[All Fields] OR "cancerization"[All Fields] OR "cancerized"[All Fields] OR "cancerous"[All Fields] OR "neoplasms"[MeSH Terms] OR "neoplasms"[All Fields] OR "cancer"[All Fields] OR "cancers"[All Fields]) AND ("Patient Reported Outcome Measures"[MeSH Terms] OR ((("Patient Reported Outcome Measures"[MeSH Terms] OR ("patient"[All Fields] AND "reported"[All Fields] AND "outcome"[All Fields] AND "measures"[All Fields]) OR "Patient Reported Outcome Measures"[All Fields] OR ("patient"[All Fields] AND "reported"[All Fields] AND "outcome"[All Fields]) OR "patient reported outcome"[All Fields]) AND "measure*"[All Fields]) OR ("patient report*"[All Fields] AND "outcome*"[All Fields] AND "measure*"[All Fields]) OR "prom"[All Fields] OR "PROMIS"[All Fields]))) AND (("response"[All Fields] OR "responses"[All Fields] OR "responsive"[All Fields] OR "responsiveness"[All Fields] OR "responsivenesses"[All Fields] OR "responsives"[All Fields] OR "responsivities"[All Fields] OR "responsivity"[All Fields]) OR "rate"[All Fields]))) AND (2000:2022[pdat]).
